# A systematic review of pesticide exposure, associated risks, and long-term human health impacts

**DOI:** 10.1016/j.toxrep.2024.101840

**Published:** 2024-11-30

**Authors:** Chander Shekhar, Reetu Khosya, Kushal Thakur, Danish Mahajan, Rakesh Kumar, Sunil Kumar, Amit Kumar Sharma

**Affiliations:** Department of Animal Sciences, Central University of Himachal Pradesh Shahpur Campus, Kangra 176206, India

**Keywords:** Pesticide, Health risks, Long-term effects, Pesticide regulation, Safety protocols

## Abstract

Pesticides are widely used to control pests, but their widespread use raises concerns regarding potential health risks for humans. There are several routes through which pesticides can be ingested, inhaled, and absorbed, resulting in acute and long-term health consequences. This systematic review synthesizes the available evidence regarding the health risks and long-term effects of pesticide exposure, with a particular focus on epidemiological and toxicological studies. A systematic review was conducted by searching scientific databases i.e. Scopus, and Web of Science for peer-reviewed articles published between 2000 and 2024. Studies were selected based on their focus on pesticide exposure, health risks, and long-term effects. Meta-analysis was conducted where sufficient homogeneity of outcomes allowed. This review identified consistent associations between chronic pesticide exposure and non-communicable diseases, including cancer, neurological disorders, and endocrine disruptions. An increased incidence of respiratory issues and neurodegenerative diseases was often associated with occupational exposure to pesticides. People exposed for a prolonged or high intensity time period, particularly agricultural workers, were more likely to experience long-term health effects. There are a number of factors that influences the ability to draw definitive conclusions, including variations in pesticide types, exposure levels, and health outcomes. Chronic exposure to pesticides presents significant health risks, particularly for individuals in high-exposure environments like agriculture. While evidence indicates strong associations with several long-term health conditions, additional research is necessary to elucidate dose-response relationships and mechanisms of action. This review underscores the necessity for enhanced regulatory measures and improved safety protocols to mitigate pesticide-related health risks.

## Highlights

• Pesticide exposure raises health risks, including cancer and neurological issues.
• Occupational exposure raises health risks due to prolonged pesticide contact.
• Long-term pesticide effects from low-dose exposure through inhalation and ingestion.
• Regulations and safety protocols are key to reducing health risks from pesticides.

## Graphical Abstract

**Figure:**
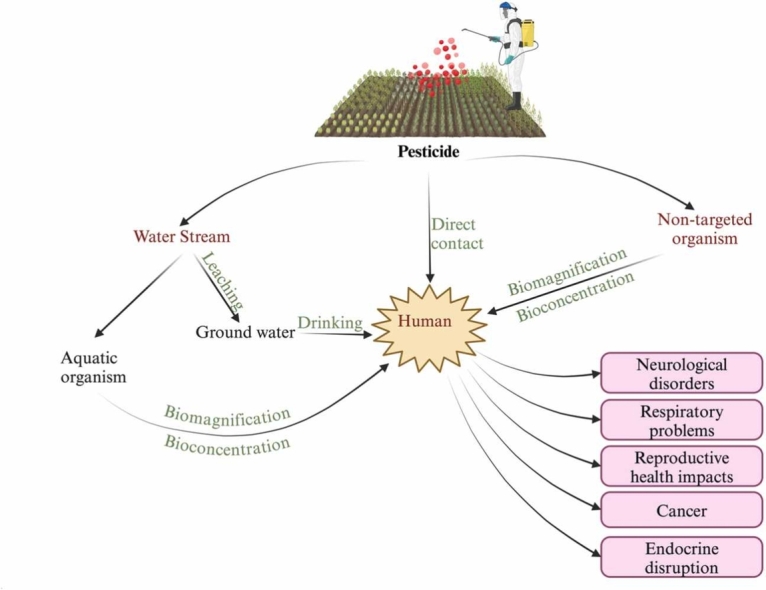

## 1 Introduction

The term "pesticide" is derived from the Latin words “pestis,” meaning plague, and “caedere,” meaning to kill. This term broadly encompasses a range of chemicals used to manage and control pests, which include weeds, plant pathogens, insects, molluscs, nematodes (roundworms), birds, fish, mammals and microbes that compete with humans for food, destroy property, spread or help carry or spread diseases or are seen as a nuisance [174]. Pesticides are employed in numerous contexts, from agriculture, where they protect crops from pests, to public health initiatives aimed at controlling disease vectors like mosquitoes. The diverse categories of pesticides include herbicides, insecticides, fungicides, rodenticides, and more, each targeting specific types of pests or functions. Today, a variety of pesticides are available, based on the needs of modern society. However, there are three primary classifications into which pesticides are often divided: Pesticides that target specific pest organisms, pesticides that target pest entrance points and pesticides that are primarily focused on the chemical makeup of the pesticide [70].

The pesticide is further divided into a few smaller classes according to factors such as its method of action, origin, target range, and kind of pesticidal formulation [70]. Based on their toxicity, pesticides were categorized by the World Health Organization into four categories: extremely dangerous, highly dangerous, moderately dangerous, and slightly risky. [169]. Pesticides are used extensively in modern agriculture because they are a cost-efficient and efficient technique to improve produce quality and quantity by protecting the crops from various pests and maintaining food security for the world's expanding population. An estimated 2 million tons of pesticides are used worldwide each year; China is the country consuming the most, followed by the United States and Argentina, whose use is growing at a rapid rate [135].

Historically, the use of substances to control pests dates back thousands of years. Early methods involved natural substances like ashes and salt. Ancient civilizations, such as the Sumerians and Chinese, used elemental compounds like sulphur, mercury, and arsenic to manage pest populations [31]. These early practices laid the foundation for modern pest control strategies. The 20th century marked a significant shift with the advent of synthetic pesticides [58]. When Dichlorodiphenyltrichloroethane (DDT) and Benzene hexachloride (BHC) were introduced to India in 1948 to fight malaria and locust infestations, respectively, the country began to use pesticides. India built a factory in 1952 to manufacture the pesticides DDT and (BHC). In 1958, India produced around five thousand metric tons of pesticides. Approximately 145 pesticides are currently approved for use, and 85,000 metric tons are generated each year.

These substances are widely used, although this has had a number of detrimental short- and long-term effects [85]. When parathion-contaminated wheat flour killed over 100 people in Kerala in 1958, it was the first known occurrence of pesticide poisoning in India. Many reported incidents of pesticide poisoning have occurred since the Bhopal tragedy [51]. Despite the fact that most contemporary formulations are safe for non-target species, a wealth of theoretical and experimental evidence shows that pesticide residues can have long-term detrimental impacts on animal and human health as well as the stability of ecosystems [69]. While there are certain benefits to using pesticides, such as increased productivity, their careless and excessive usage also has a negative effect on human health and the environment. The current state of pesticide use does not seem to be adequate; even so-called safe pesticides are beginning to exhibit long-term negative impacts, and issues like bio-accumulation and bio-magnification are becoming more and more problematic.

The so-called benefits of pest control have already had an impact on almost every region of the globe, every living thing that inhabits it, and every organism that is yet to exist [120]. Since pesticides don't have species-specific modes of action, worries have been expressed over the environmental dangers that come with being exposed to them through a variety of channels (e.g., residues in food and drinking water). These risks can be short-term (e.g., headaches, nausea, dizziness, and skin and eye irritation) or long-term (e.g., cancer, asthma, and diabetes). However, because a number of factors are involved (e.g., duration and intensity of exposure, kind of pesticide (in terms of toxicity and persistence), and environmental features of the impacted areas), it is challenging to determine the exact nature of these risks. Since most diseases have several causes and there are no groups in the human population who are entirely immune to pesticides, public health assessments are significantly more difficult [73].

The primary concerns arise from both direct and indirect exposure. Direct exposure can occur during the application of pesticides, potentially causing immediate health effects such as respiratory problems, skin irritation, or headaches. Indirect exposure happens when pesticide residues remain on food, leach into water supplies, or accumulate in the environment, eventually affecting human health through ingestion or contact. A significant issue associated with pesticide use is bioaccumulation, where these chemicals build up in the body over time, especially when exposure is frequent or prolonged [120]. This accumulation can lead to more severe health problems, as pesticides are persistent in the environment and can magnify through the food chain, a process known as biomagnification. As a result, the health impacts can be amplified at higher trophic levels, including humans. Almost always, pesticide residues are found in combinations with other substances.

Despite growing worries regarding their safety, the toxicological consequences of low-dose pesticide combinations on human health are largely unknown. Certain organophosphates can become more harmful when combined with other organophosphates or when exposed to organochlorines in the past. Specific interactions are also covered, including the promotion of organophosphate-induced delayed polyneuropathy, the cumulative toxicity of organophosphates and organochlorines leading to estrogenic consequences, and pesticides functioning as endocrine disruptors [57], [105], [106]. Due to long-term directly and indirectly exposure to pesticides are responsible for various health problems, including reproductive disorders, respiratory diseases, cardiovascular problems, gastrointestinal disturbances, and neurological conditions.

The chronic nature of these health effects often becomes evident only after years of exposure, emphasizing the need for stringent regulations and better management practices. In summary, while pesticides play a crucial role in modern agriculture and public health, their use carries significant risks that must be managed carefully. The balance between leveraging their benefits and mitigating their harmful impact on the health of human and the environment is critical for sustainable pest management [136].

This study aims to systematically categorize the various classes of pesticides and to summarize the existing literature on human impacts associated with their exposure, focusing on associated diseases. This review will provide a comprehensive overview of the relationship between pesticide classes and human health, serving as a valuable resource for researchers, policymakers, and public health officials indicating that further research is required for developing standardized methodologies for assessing pesticide exposure and investigating the efficacy and safety of the alternative pest control methods that could provide insights into reducing reliance on harmful pesticides.

## 2 Methodology

This study is based on a systematic review methodology and asked “What are the effects of pesticide residue on human health? The objectives of the study are given below:

• Reviewing relevant research and reviewed articles about the effects of pesticides on humans that have been published in reputable journals.
• Effects of Bioaccumulation and Biomagnification in the transfer of pesticide residue.
• Classification of various effects of pesticides on human beings.
• To summarize the main findings conclusions and suggest further research paths.

For this study, a variety of important scientific databases were searched, including “ScienceDirect, PubMed/MEDLINE, Scopus, Web of Science, ResearchGate and Google Scholar”. The databases include an abundance of information that has been consulted and is related to toxicology, human health and environmental research. Relevant keywords such as pesticides, AND classification, AND toxicity, AND human health will be included in the search strategy. Boolean operators (AND, OR) were utilized in order to narrow and widen search queries. Authors determine what is included and what is not. The studies that were considered were those that were released in the years 2000–2024. For the purposes of this study, a number of reviews, and the research papers that have been published in journals were included. Numerous researches have been conducted on the impacts of pesticides (such as carbamate and organophosphate insecticides) on both terrestrial and aquatic life, as well as how these pesticides may change their behaviour.

Articles written in languages other than English (unless an English translation was available) and research not directly connected to the classification of pesticides and toxicity of pesticides to human being were excluded. Editorials, opinion articles, and conference abstracts were not selected. Two independent authors conducted a preliminary assessment of abstracts and titles as part of the selection process. In order to determine the full-text's criteria for inclusion and excluded, a full-text screening will be performed. Articles with relevant information are included while non-qualifying articles were disqualified. Once the data extraction process is finished, the extracted data can be verified by generating a standard format. The study had a number of data items, such as author's name, year of publication, study design, pesticide, transfer of pesticide residue and effects on human health [137].

The narrative-based methodology was used to arrange and summarize the study's findings. Various significant issues were found and explored, including the classification of pesticides and their effects on humans. The included papers were evaluated and reported using a standardized tool, the PRISMA-S (Preferred Reporting Items for Systematic Reviews and Meta-Analyses literature search extension) checklist for systematic reviews, in accordance with previously published procedures [78], [126].

These approaches include three main steps:

**Identification:** In this step firstly the research gap is found. Then the literature according to the topic is researched.

Record identified: 960

**Screening:** The data related to the topic is extracted and then a deep screening is conducted. In screening, all the irrelevant data is removed from the study like erratum, editorials, notes, data, conference papers and letters and only the authentic data is kept and studied thoroughly.

Excluded: 200

**Included:** All the evaluated data is compiled in the form of a review paper

**Fig. 1:**
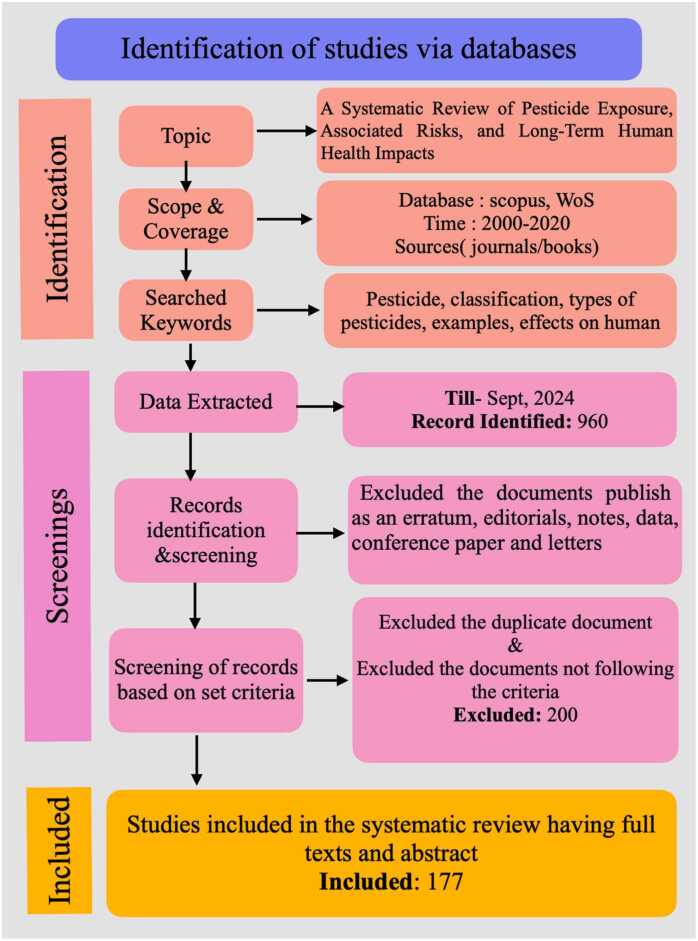
Systematic methodology of the current study.

Included: 177 (Fig. 1).

## 3 Classification of pesticide

The classification of pesticides is essential for understanding their diverse applications, effectiveness, and safety. Pesticides are classified based on a number of factors, including their toxicity or potentially harmful effects, their application or purpose, their chemical structure, their mode of action, function, their formulations, and source of their origin [1]. Each classification method provides valuable insights into how pesticides function, their environmental impact, and the best practices for their application. Understanding the chemical composition of pesticides helps determine their efficacy and behaviour in the environment. This includes differentiating between major chemical groups such as organochlorines, organophosphates, carbamates, and pyrethrins/pyrethroids. In addition, pesticides can be classified according to their specific application. Another critical classification criterion is how pesticides interact with pests and their environment, including systemic versus contact effects. Finally, the physical state of pesticides whether gaseous, liquid, or solid affects their application methods and effectiveness. These classification systems collectively provide a framework for selecting and using pesticides effectively while managing potential danger to the health of humans and the environment. The classifications of the pesticides are majorly done into three major types:

### 3.1 Pesticide classification based on application

**Fig. 2:**
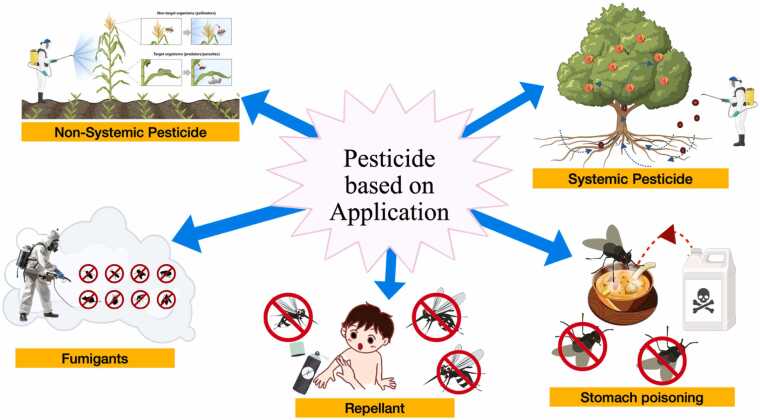
Pesticide based on Application.

The mode of entry describes how pesticides interact with or penetrate the target organism. Understanding these methods is crucial for effective pest control and safe application. Pesticides can be classified based on how they come into contact with pests, and these modes of entry include systemic, contact, stomach poisons, fumigants, and repellents (Fig. 2).

**Systemic Pesticides**: They are applied and absorbed into the plant or animal and are distributed throughout its tissues. After application, pesticides penetrate through the leaves, trunk and roots of plants as well as via the digestive system and bloodstream of other organisms including humans [162]. When they are applied to the plants, pesticides enter throughout the tissues of the plant, including those not directly treated. This makes them effective against pests that feed or live on the plant tissues. In non-plant organisms, systemic pesticides can also be ingested, inhaled, absorbed through the skin and then circulated through the body to affect pests.

**Contact pesticides**: Contact pesticides act directly on the pests when they come into contact with them. They do not require ingestion or systemic distribution to be effective. Contact pesticides are applied to the surfaces where pests are likely to come into contact, such as plant leaves, soil, or surfaces where pests are active. These pesticides typically work by disrupting the pest’s physiological functions or causing immediate damage upon contact [20]. They are often used for rapid pest control and are effective in managing pests on the exterior of plants and surfaces.

**Stomach poisons**: Stomach poisons function by being ingested by the target pest. Once consumed, these pesticides disrupt the digestive processes or internal functions of the pest like rapid intoxication, lack of coordination, paralysis and leading to its death [107]. Stomach poisons are often formulated as bait or feed additives, making them particularly useful for controlling pests that feed on plant material or stored products. The effectiveness of stomach poisons depends on the pest's feeding habits and the pesticide's ability to be consumed in sufficient quantities.

**Fumigants:** Fumigants are gaseous pesticides that penetrate through the air and soil, reaching pests in enclosed spaces or within the soil. These chemicals vaporize and diffuse into the environment, targeting pests that are hidden or not directly exposed to surface treatments. Fumigants are used in various settings, including agriculture, storage facilities, and buildings, to control pests such as insects, nematodes, and fungi. The gaseous nature of fumigants allows them to infiltrate and kill pests in hard-to-reach areas, offering comprehensive pest management solutions [39].

**Repellents**: Repellents are designed to deter pests from coming into contact with or feeding on treated surfaces or plants. Unlike other pesticides that kill pests, repellents function by creating an unpleasant or irritating effect that drives pests away. They include mating disruption, pheromone antagonists as chemical communication inhibitors, pheromones and plant-based volatiles, attractant-and-kill, and push–pull strategies [125]. They can be applied to plants, organisms, or surfaces and are used to prevent pest infestations rather than eliminate existing ones. Repellents are commonly used in both agricultural and household settings to protect crops, stored goods, and living spaces from pest damage.

Each application of pesticides plays a unique role in pest management strategies, and selecting the appropriate method depends on the type of pest, the environment, and the desired outcome of the treatment. Understanding these applications helps in devising effective pest control plans while minimising potential risks to non-target organisms and the environment (Table 1).

**Table 1:** Pesticide classification based on application.

**Type of Pesticide**	**Function**	**Example**	**References**
Systemic Pesticides	These are pesticides which are absorbed by plants or animals and transfer to untreated tissue	acetamiprid, boscalid or difenoconazole	[84]
Contact pesticides	It acts on target pests when they come in contact	Acephate, carbaryl, fipronil, pyrethrins, pyrethroids	[27]
Stomach poisons	It enters the pest’s body through their mouth and digestive system	Malathion	[107]
Fumigants	Pesticides which acts or may kill the target pests by producing vapour and enter pest’s body through tracheal system.	Phosphine	[39]
Repellents	Repellents do not kill but distasteful enough to keep pests away from treated area. They also interfere with pest’s ability to locate crop.	Methiocarb	[125]

### 3.2 Classification based on pesticide function and pest organism they kill

Pesticides are made to control different kinds of pests and safeguard crops, buildings, and people's health. They are divided into groups according to the particular roles they play and the organisms they kill (Table 2).

**Table 2:** Classification of pesticides on the basis of their function, pest organisms they kill and the case studies of their effect.

**Type of Pesticide**	**Function and Target Pest**	**Example**	**Impact**	**Refrences**
Acaricides	Kill mites and ticks or to disrupt their growth or development	DDT, dicofol, chlorpyrifos, permethrin, etc	Baseline exposure to organochlorine (OC) pesticides can leads to cognitive impairment and dementia in the elderly	[80]
Algicide	Substances that used to kill or inhibit algae	Copper Sulphate, diuron, oxyfluorfen, etc	Liver damage and wilson’s disease	[101], [153]
Antifeedants	Chemicals which prevent an insect or other pest from feeding	Chlordimeform, azadirachtin, etc	In children it leads to toxic encephalopathy and Reye's-like syndrome. In an elderly male patient it leads to vomiting, seizures, metabolic acidosis and toxic encephalopathy.	[96]
Avicides	Chemicals that are used to kill birds	Strychnine, fenthion, etc	Initial symptoms are tightness and twitching of the muscles, agitation and hyperreflexia, stiffness of the body, lockjaw, frothing of the mouth, and cessation of respiration and later Tetanus-like attacks appear every 10–15 min	[112], [168]
Bactericides	Compounds that isolated from or produced by a microorganism or a related chemical that is produced artificially, which are used to kill or inhibit bacteria in plants or soil	Streptomycin, tetracycline, etc	There were no such sevier effects seen.	[165]
Bird repellents	Chemicals which repel the birds	Diazinon, methiocarb, etc	Impact on non-target native birds, such as robins and tomtits	[38]
Chemosterillant	Chemicals that renders an insect infertile and thus prevent it from reproducing.	Diflubenzuron, zinc gluconate	Adverse effects related to the haematopoietic system include an excess of methaemoglobin (methemoglobinemia) and/or sulfhemoglobin (sulfhemoglobinemia) in the blood.	[17]
Desiccants	Act on plants by drying their tissues	Boric acid	In some cases it can lead to cardiac arrest	[104]
Fungicides	Chemicals which are used to prevent, cure eradicate the fungi.	Cymoxanil, thiabendazole, Bordeaux mixture	Carcinogenic effects, tumor formations in liver and thyroid tissues	[53], [156]
Herbicide softener	A chemical that protect crops from injury by herbicides, but does not prevent the herbicides from killing weeds.	Benoxacor, cyometrinil	Benoxacor, a safener with the highest potential for toxicity, was found to be highly toxic, particularly in the liver.	[143]
Herbicides	Substances that are used to kill the plants, or to inhibit their growth or development.	Alachlor, paraquat, 2,4-D	Ingestion can lead to nausea, vomiting, abdominal pain, and loss of consciousness.	[75]
Insect attractant	A chemical that lures pests to trap, thereby removing them from crops animals and stored products	Gossyplure, Gyplure	Sometimes it can lead to severe crop damage.	[108]
Insect growth regulator	A substance that works by disrupting the growth or development of an insect	Diflubenzuron	It can kill various non targeted organism	[160]
Insecticides	A pesticide that is used to kill insects or to disrupt their growth or development	Azadirachtin, DDT, chlorpyrifos, malathion, etc.	Its consumption can lead to vomiting, diarrhea, and abdominal pain.	[25]
Larvicides	Inhibit the growth of larvae	Methoprene, Pyriproxyfen	No effects were seen on humans	[64]
Lampricides	Target larvae of lampreys which are jawless fish like vertebrates	Nitrophenol, 3-trifluoromethyl−4-nitrophenol	Non-toxic to mammals and pose no threat to wild life	[61]
Mammal repellent	A chemical that deters mammals from approaching or feeding on crops or stored products	Anthraquinone	Effects on non-target organism and may lead to death in some cases	[34]
Mating disruptors	Interfere with the way that male & female insects locate each other using airborne chemicals, thereby preventing them from reproducing	Disparlure, gossyplure, etc.	Exposure to non-target organisms causes altered hormone levels, importantly gonadal hormones, resulting in changed reproductive characteristics.	[138]
Molluscicides	Substances used to kill slugs and snails.	Metaldehyde, thiadicarb, etc.	The patients experience gastrointestinal symptoms, neurologic complications, seizures amd some time suicidal attempts	[149]
Moth balls	Stops any damage to cloths by moth larvae	Dichlorobenzene	Its ingestion causes encephalopathy, psychomotor retardation, flat affect, and cognitive decline	[103]
Nematicides	Chemicals which are used to control nematodes	Carbofuron, chlorpyrifos, methyl bromide, etc.	• Highly lethal to mammals, birds, fish, and wildlife. • Inhibits acetyl-cholinesterase and butyrylcholinesterse activity. • Associated with endocrine disrupting, reproductive disorders, cytotoxic, and genotoxic abnormalities in humans.	[97]
Ovicides	Inhibit the growth of eggs of insects and mites	Dicofol, Avermectin	Non-toxic to mammals	[41], [154]
Piscicides	Acts against fishes	Rotenone	Its consumption can lead to respiratory arrest, cardiac arrest and often lead to death	[152]
Plant growth regulators	Substances alters the expected growth, flowering or reproduction rate of plants	2,4-D, gibberellic acid, etc.	Gibberellin can potentially upregulating AMPK and reducing fertilization potential in human semen samples.	[173]
Rodenticides	Substances used to kill rats and related animals	Strychnine,Warfarin, zinc phosphide, etc.	Its ingestion can lead to kidney damage, liver enzymes, pancreatitis, and myocarditis, respiratory distress syndrome, liver, renal failure and often lead to death	[176]
Silvicides	Use in thinning of over dense woody vegetation	Cacodylic acid, monosodium methanearsonate	Increase of arsenic level in urine of worker applying silvicides	[8], [151]
Synergists	A chemical enhances the toxicity of a pesticide to a pest but that is not by itself toxic to pest	Piperonyl butoxide	Piperonyl butoxide has low acute toxicity, with potential for anemias, liver damage, and metabolic enzyme imbalances	[166]
Termiticides	Kill termites	Fipronil	It can lead to altered sensorium, seizures, and jaundice.	[65]
Virucide	An agent having capacity to destroy an inactivate viruses	Ribavirin	It can higher peak C-reactive protein and lactate dehydrogenase levels and higher mortality rates.	[22]
Miscellaneous	It can act as a insecticides and rodenticides	Aluminium phosphide, sodium cyanide	It can causing severe symptoms including vomiting, an upset stomach, and death. It can also lead to cardiac myocytes, fluid loss, and adrenal gland damage	[94], [121]

### 3.3 Classification based on chemical composition of pesticides

**Fig. 3:**
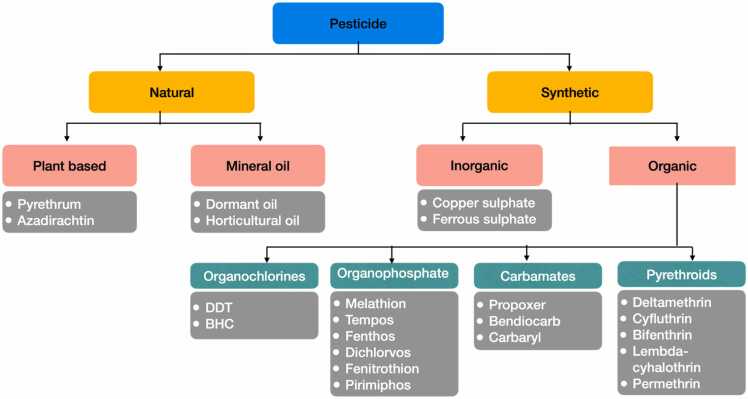
Pesticide based on Chemical Composition.

The most popular and practical way to categorize pesticides is by their active components and chemical makeup. These types of classification give detail information about the chemical, physical, and effective characteristics of the individual pesticides. Understanding the chemical and physical characteristics of pesticides can be beneficial in determining the application method, application rates, and any essential safety precautions during application. Based on their chemical composition, pesticides are divided into four main classes: organochlorines, carbamates, pyrethroids, pyrethrin, and organophosphorus. Pesticides are classified according to a complicated system of chemicals. Modern insecticides are often made of organic substances. They consist of both synthetic and plant-based insecticides (Fig. 3).

**Organochlorines (OC):** Pesticides having five or more chlorine atoms bonded to organic molecules are called organochlorines or polychlorinated hydrocarbons. OC are synthetic compounds with aliphatic and aromatic chains used in various industries like polymers, solvents, pesticides, and plasticisers. These are some of the earliest types of pesticides ever developed and used in agricultural and public health. Most of them were often used as pesticides to control a range of insects, and most have a persistent residual effect on the environment. They are able to manage practically any type of pest, such as fungi, rodents, and insects [71]. These pesticides have the potential to cause neurological disruption in insects, resulting in convulsions, paralysis, and ultimately death. The most prevalent pesticides that fall within this category are lindane, aldrin, dieldrin DDT, endosulfan and chlordane. Even though the majority of industrialised nations, including the United States, forbade the production and use of DDT many years ago, most tropical developing [51]. OC has great toxicity, slow degradation in nature and are high power of bioaccumulation [67]. **Examples:** Aldrin, Chlordane, DDT, Dieldrin, Endrin, Heptachlor, Lindane, Mirex, Methoxychlor, and Toxaphene etc.

**Organophosphates (OP):** Organophosphate pesticides (OPPs) encompass diverse organic compounds containing a phosphate group in their structure, with numerous applications. OP pesticides are regarded as broad-spectrum insecticides capable of controlling a broad variety of pests. They are typified by fumigant poison that leads to nerve poisons, stomach poison, and contact poison. Additionally, these pesticides are slow to develop pest resistance, biodegradable, and cause little harm to the environment. OPPs are comparatively more poisonous to vertebrates and invertebrates than cholinesterase inhibitors because they produce a persistent acetylcholine (ACh) neurotransmitter cover over a synapse [90]. Acetylcholinesterase (AChE) is inhibited by OP chemicals, which causes acute toxicity. Many people may develop intermediate syndrome, which has the potential to cause respiratory paralysis and even death. Consequently, the failure of nerve impulses to pass the synapse results in a fast twitching of voluntary muscles, which ultimately causes paralysis and death. Potentially, phosphorylation of AChE leading to ACh buildup, overstimulation of cholinergic receptors, and resultant clinical symptoms of cholinergic toxicity is a mechanism of toxicity shared by all organophosphate anticholinesterases [119]. Example: Malathion, parathion, diazinon, fenthion, chlordecone, Imidacloprid, dichlorvos, chlorpyrifos, ethion, nerve gases (soman, sarin, tabun, VX), ophthalmic agents (echothiophate, isoflurophate), and antihelmintics (trichlorfon) etc [113].

**Carbamates (CM):** CM are organic compounds derived from carbamic acid (NH_2_COOH). Carbamides and organophosphates share a similar structural makeup. however, the main difference lies in their composition. OP contain organophosphate, while CM consist of carbamic acid. CMs are generally used industrially and domestically. They have acute toxicity toward insects and mammals due to their inhibitory action on AChE and other esterases [49]. On rare occasions, they are utilised as stomach and contact poisons, fumigants, and both. They can be easily degraded in the environment [174]. CM is connected to an increase in immune system-related disorders, such as cancer, autoimmune diseases, and hypersensitivity reactions. Through direct immunotoxicity, endocrine disruption, and esterase activity inhibition, these pesticides may exacerbate immunological dysfunction. CM pesticides have the potential to cause gene alterations that affect immunological tolerance and immune control, as well as to start, intensify, or enable detrimental immune responses. The research evaluates the data linking immune system dysregulation to CM pesticide exposure and its possible associations with a range of malignancies, allergies, autoimmune conditions, and infectious illnesses [36]. Several widely used pesticides, including aminocarb, carbofuran, propoxur, and carbaryl, are included in this group. **Example**: Carbaryl, Carbanolate, Propoxur, Carbofuran, bendiocarb, Butylate, Cycloate etc.

**Pyrethroids:** Pyrethroid insecticides consist of natural pyrethrins extracted from pyrethrum flowers and their synthetic derivatives are called pyrethroids [92]. Synthetic pyrethroids can be synthesized by duplicating the structure of natural pyrethrins. Compared to natural pyrethrins, they have longer residual effects and are more stable. Pyrethrins are grinded to form active ingredients like Pyrethrins I and II, which make up the majority of the active ingredients, with minor amounts of the related cinerins and jasmolins [174]. The compounds are used in various formulations, through which they cause multiple toxicity effects. For example, pyrethroids deltamethrin and cis-methrin are known to trigger alteration to the membrane conductance in skeletal muscle and non-myelinated nerve fibre preparations from rats were evaluated [42]. Synthetic pyrethroid-containing pesticides provide a minimal threat to birds and mammals but are very toxic to fish and insects. Most artificial pesticides are not very long-lasting and degrade easily in the presence of light. These are regarded as some of the safest pesticides to apply to food. The most widely used synthetic pyrethroid insecticides are permethrin and cyclomethrin. Examples: Dimethrin, Cypermethrin, Tetramethrin, Cyclethrin, Furethrin, Fenevelerate, Alphamethrin, Pyrethrin, Bonthrin, Decamethrin, Allethrin.

### 3.4 Other Classes of Pesticides

Pesticides can be further divided into a number of smaller classifications according to their toxicity and formulation. Every classification sheds light on the properties, origins, application preparation, and possible risks of these compounds. Knowing these minor classes makes it easier to choose the right pesticide for a given pest problem while taking the effects on human health and the environment into account.

**Based on types of pesticide formulation**: Generally speaking, "raw" or unformulated pesticide chemicals are unsuitable for pest management. Due to their instability, these concentrated chemicals and active ingredients may be challenging to handle and transport, and they may not mix well with water. To enhance application efficiency, safety, handling, and storage, manufacturers incorporate inert materials like solvents and clays. The purpose of inert ingredients is to act as a carrier for the active component, as they do not have any pesticidal effect. When inert and active ingredients are mixed, they are referred to as a pesticide formulation [117]. To make the pesticide easier to measure, mix, and apply, as well as safer for the user, inert materials (water, petroleum solvent, wetting agents, spreaders, stickers, and extenders) are components added to the active ingredient. Chemicals called active ingredients are designed to fight specific pests. The effectiveness of a pesticide formulation can be increased by mixing two or more different pesticide groups together. To make pesticide formulations, technical-grade insecticides are mixed with inert diluents and auxiliary compounds. There are three main classes of pesticide formulations: liquids, solids, and gases (Table 3).

**Table 3:** Classification of pesticide based on their formulation.

Physical State	Active substance	Examples	Function	Target organism	References
Solid	Bait	Maxforce FC, Niban, Amdro etc	These are the active components combined with an attractant or pest food.	Birds, ants, slugs, snails crickets and grasshoppers.	[35], [87]
	Dust	Deltadust, Ficam D, Drione, Sevin D, Malathion D	These are combinations of an active substance and a carrier material that have been finely powdered. The purpose of dust formulations is to be applied directly, without additional mixing. i.e., talc, clay, and volcanic ash.	Spot treatment, Animal powder, Seed treatment.	[129]
	Granules	Dursban G, Talstar G	The active ingredient is mixed with various inert clays to form particles of various sizes. size of granules usually ranges from 20 to 80 mesh	Mosquito larvae, weed control	[55]
	Pellets	Metaldehyde, ferric phosphate	Inert material containing active ingredient like granules, but has more uniform shape and weight.	rodents, slugs	[40]
	Soluble powder	Cartap hydrochloride, nitenpyram	Dry powder which dissolves in water to spray solution.	insects & weed control	[129]
Liquid	Aerosols (A)	Wasp Freeze, ULD-BP−50, Ultracide, Ultraguardian	Usually contain small amount of active ingredient and a petroleum solvent.	Insecticides or mosquito control.	[12]
	Emulsifiable concentrate	Chlorpyrifos EC Cypermethrin EC	Contains active ingredient, petroleum solvent and emulsifiers	insect, disease and weed control	[21]
	Flowable	Carbaryl AF	Finely ground particles suspended in an inert liquid carrier	insect, fungal disease and weed control	[95], [157]
	Gel	Sepiolite	Semi liquid emulsifiable concentrate	Herbicides and insecticides	[89]
	Solution	Premise SC, Termidor SC, Bora-care	Active ingredient dissolved in liquid.	weed control	[129]
	Ultra-low volume concentrate (ULV)	Thidiazuron diuron	Liquid with very high concentration of active ingredient designed to be used as is or slightly diluted in ULV equipment.	agricultural, forestry, ornamental, and mosquito control	[83]
Gas	Fumigants	Phosphine, Phostoxin, Methyl bromide	Pre-weighed amount of WP or SP formulation in a special plastic bag which dissolves in spray tank and releases contents.	Greenhouses, mushroom houses, graineries. Pre-plant soil treatment for soil borne pests	(Sande et al., 2011)

**Based on toxicity of pesticides:** According to World Health Organization (WHO), the primary basis for classification, as these determinations are standard procedures in toxicology, is the acute oral and dermal toxicity to the rat. A chemical will always be categorized in the more restrictive class if its oral LD50 value suggests a different class than its dermal LD506 value does. In this manner, the classification differentiates between each pesticide's more and less harmful versions. As per [169], it is dependent on the technological compound's toxicity as well as its formulations (Table 4).

**Table 4:** Classification of pesticide based on toxicity (World Health Organization et al., 2002).

WHO class	Toxicity level	LD50 for the rat (mg/kg body weight)		Examples
		Oral	Dermal	
Ia	Extremely hazardous	< 5	< 50	Parathion, Dieldrin, Phorate
Ib	Highly hazardous	5–50	50–200	Aldrin, Dichlorvos
II	Moderately hazardous	50–2000	200–2000	DDT, Chlordane
III	Slightly hazardous	> 2000	> 2000	Malathion
IV	Unlikely to present acute hazard in normal use	> _5000		Carbetamide, Cycloprothri

## 4 Transmission of pesticide residue

Pesticides are chemical compounds or mixtures of dangerous substances used by humans to control disease-carrying insects and boost crop protection against pests. Because of its wide use, it may be cause danger to non-targeted plants and animals. When pesticides are sprayed or dispersed over entire crop fields, runoff can carry the chemicals into aquatic environments, and wind can transport the toxins to farms, grazing areas, and populated areas, endangering other animals [131]. Numerous animal-based foods have been found to contain pesticides residue, which is dangerous for both human health and food safety [82]. Pesticides are extremely harmful to aquatic life, including fish and snails.

Pesticides also cause neurological impairment in freshwater organisms. Pesticides have the potential to seriously alter the biochemistry and histology of freshwater species by generating an excessive amount of reactive oxygen species (ROS) [131]. The aquatic environment, the ecosystem, and human health are all negatively impacted by the produced wastewater that is tainted with pesticides. Overuse of pesticides results in chemically polluted wastewater, which is bad for the ecology, the aquatic environment, and people's health [122]. While some pesticides are biodegradable and others are not, the biodegradable ones take a very long time to breakdown. The amount of time the pesticide is left in the soil determines the rate at which it breaks down, and other environmental factors may additionally have an impact [7].

Between these times there is a high chance of the pesticide to disperse in the environment. One of the major factors for the dispersion of the pesticide residue is by the rainfall [62]. Based on research, the maximum amounts of pesticides mostly from agricultural sources in roof runoff and rain happen during and right after application periods. For example, for single rain events and annual loads, atrazine peaks at 903 ng/L and 13,900 ng/(m² year), alachlor at 191 ng/L and 5900 ng/(m² year), and R-dichlorprop at 106 ng/L and 5100 ng/(m² year). Furthermore, significant amounts of these substances that are washed into the environment can end up in groundwater, particularly if roof runoff penetrates deeply porous underneath layers.

Occasionally, they can even enter adjacent waterbodies like rivers, and lakes directly [15]. Water samples from 21 wells and two springs in a Pennsylvanian watershed that is primarily used for agriculture were examined for the presence of 11 pesticides, NO_3_, Cl, and PO_4_. Only atrazine was commonly found among the pesticides reported by a farm use study, but at very low amounts ranging from 3 ng/L to 1.11 μg/L. During a three-sampling sequence designed to cover important groundwater recharge times, atrazine was detected at least once in 74 % of agricultural wells [118]. Due to the continuous contamination of the water exposure of the pesticide residue is increasing with living organism and leading to the bio-transfer of these residues by the help of biomagnification and bioaccumulation [24].

Pesticides can enter fish through two primary routes: directly from the environment (water, sediments) via the gills or skin, and from their food sources through the alimentary canal. The process by which a pesticide accumulates in fish from these sources is termed bioaccumulation. This accumulation occurs through two mechanisms: bioconcentration and dietary accumulation. Bioconcentration refers to the buildup of a pesticide directly from the abiotic medium, while dietary accumulation involves the uptake of pesticides from ingested prey.

Biomagnification, also known as biological magnification or bioamplification, describes the progressive increase in pesticide concentration at each successive trophic level within a food chain [76], [134]. This phenomenon results from the accumulation of pesticides in organisms at lower trophic levels, which are then consumed by organisms at higher levels, leading to higher concentrations at each step [4]. Conversely, biodilution is the term used to describe a decrease in pesticide concentration at higher trophic levels. Different fish tissues and organs can accumulate varying amounts of pesticides.

**Fig. 4:**
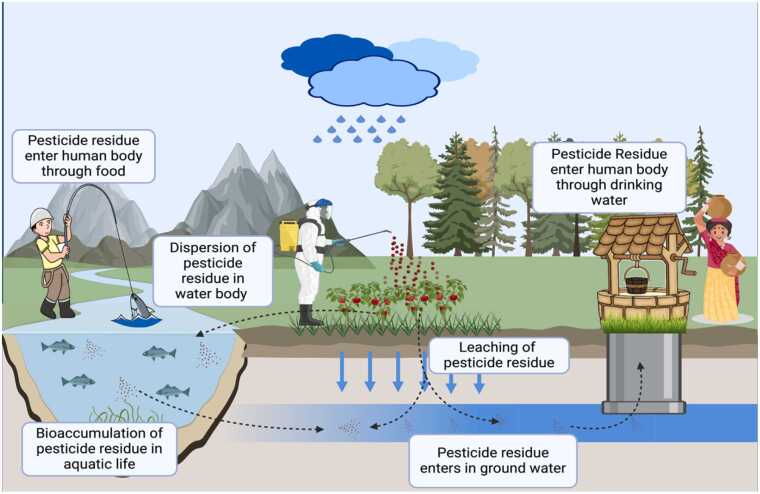
Diagrammatical Representation of Transmission of Pesticide Residue.

Metabolically active tissues such as the liver, gills, brain, muscles and kidneys typically accumulate higher concentrations of pesticides compared to less active tissues such as the skin and muscles [5], [33], [50]. Therefore, to accurately assess biomagnification and biodilution, it is essential to measure whole-body pesticide concentrations across all representative organisms within each trophic level of the food chain (Fig. 4).

## 5 Human health at risk

Pesticides are an essential instrument for safeguarding food and commercial goods, and about 1.8 billion people work in agriculture [77]. Pesticides are commonly used to manage weeds, insect infestations, and various pests in various settings. Farmers can prevent losses of 78 % (fruits), 54 % (vegetables), and 32 % (cereals), by using pesticides [159]. However, concerns about environmental risks have been raised due to their non-species-specific modes of action. Sprays of pesticides have two possible effects on non-target vegetation: they contaminate abiotic factors and non-target plants directly or drift/ volatilise from the treated area. Exposure to pesticides can lead to short-term and long-term effects, influenced by factors like time, pesticide type, and environment [73]. Since most diseases have several causes, there is no group of people in the human population that is entirely immune to pesticides, which makes public health assessments extremely difficult. An estimated 11,000 people tragically lose their lives as a result of work-related poisoning, which affects 385 million people annually, or 44 % of the world's farming population [128].

Pesticides can be directly or indirectly ingested through food, as well as through application in residential, agricultural, and work settings. Furthermore, pesticides are applied on golf courses, along major thoroughfares, and other locations where the general public may be exposed to them. Pesticides are mostly exposed to humans through the food chain, the air, water, soil, plants, and animals. Although the bloodstream carries pesticides throughout the body, Additionally, they can be expelled by the urine, skin, and air that is breathed [30]. Pesticides can enter the human body through four major routes: the cutaneous, oral, ocular, and respiratory systems. Depending on the route of exposure—dermal, oral, or respiratory—pesticide toxicity can differ (inhalation) [57]. The risk of pesticide contamination often rises with dosage (concentration) and crucial times in addition to the toxicity of the relevant chemical, as would be predicted (Table 5).

**Table 5:** A comparative account on health effects caused by different chemical classes of pesticides with their structure.

**CHEMICAL CLASS**	**Example**	**Structure**	**HEALTH EFFECT**	**REFERNECES**
Organochlorides	Dichlorodiphenyltrichloroethane (DDT) (C_14_H_9_C_l5_)		• Headache, incoordination, dizziness, vomiting, tremors, convulsions, coma, nausea, • Endocrine disruption • Carcinogenic • Inflammation of the upper respiratory tract and bronchitis, blood effects such as aplastic anemia • Cell viability	[67]
	1,1-dichloro−2,2bis (p-chlorophenyl)ethane (DDD)			
	Endrin (C_12_H8C_l6_O)			
	Dieldrin (C_12_H_8_C_l6_O)			
	Methoxychlor (C_16_H_15_C_l3_O_2_)			
	Chlordane (C_10_H_6_Cl_8_)			
	Lindane (C_6_H_6_Cl_6_)			
	Endosulfan (C_9_H_6_C_l6_O_3_S)			
	Isobenzan (C_9_H_4_Cl_8_O)			
Organophosphorus	Dimefox (C_4_H_12_FN_2_OP)		• Cholinergic Overstimulation • **S**alivation, • **L**acrimation, • **U**rination, • **D**efecation, • **G**astric Cramps, • **E**mesis • Immunotoxicity • Immunosuppression, Cancer and Hypertension • tachycardia, and paralysis	[46])
	Methyl Parathion (C_8_H_10_NO_5_PS)			
	Fenthion (C_10_H_15_O_3_PS_2_)			
	Dichlorvos (2,2-dichlorovinyl dimethyl phosphate) (C_4_H_7_Cl_2_O_4_P)			
	Trichlorfon (C_4_H_8_Cl_3_O_4_P)			
	Malathion (C_10_H_19_O_6_PS_2_)			
	Temephos C_16_H_20_O_6_P_2_S_3_			
Carbamates	Carbaryl (C_12_H_11_NO_2_)		• Impair child development and IQ • Decrease lung function Respiratory distress Difficulty breathing, respiratory paralysis • Central nervous system tumor, • Unresponsiveness • muscle fasciculations, • pinpoint pupils, diaphoresis • Cardiovascular complications **,**	[139]
	Carbanolate (C_10_H_12_ClNO_2_)			
	Propoxur (C_11_H_15_NO_3_)			
	Carbofuran (C_12_H_15_NO_3_)			
	Bendiocarb (C_11_H_13_NO_4_)			
	Butylate (C_11_H_23_NOS)			
	Cycloate (C_11_H_21_NOS)			
Pyrethroids	Dimethrin (C_19_H_26_O_2_)		• Muscle weakness, • seizures, coma • twitching, • Cardiovascular complications • Renal dysfunction, • Respiratory distress • Paranesthesia, respiratory tract, • eyes, and skin irritations	[16]
	Deltamethrin (C_22_H_19_Br_2_NO_3_)			
	Cyfluthrin (C_22_H_18_C_l2_FNO_3_)			
	Lambda-Cyhalothrin (C_23_H_19_ClF_3_NO_3_)			
	Permethrin (C_21_H_20_C_l2_O_3_)			
	Tetramethrin (C_19_H_25_NO_4_)			
Anilides/Anilines	4-(4-Chloro−2-methylphenoxy)butanoic acid- MCPB (C11H13ClO3)		• Methaemoglobinemia, Anaemia, • cardiac arrhythmia • headaches, tremor, • narcosis or coma, and • parathesis, pain,	(Levels, 2000)
	(4-Chloro−2-methylphenoxy)acetic acid- MCPA (C9H9ClO3)			
Triazines	• Atrazine (C8H14ClN5)		• Weak association with cancer • non-Hodgkin's lymphoma	[148]
	• Propazine (C9H16ClN5)			
	Terbutryn (C10H19N5S)			
	• Cyanazine (C9H13ClN6)			
Quaternary diquat	Chlortoluron (C_10_H_13_ClN_2_O)		• Epigenetic modification- Histone H3, Histone deacetylase • (HDAC) 4 and 7 • Apoptosis	[130]
	(C14H15N5O6S) metsulfuron-methyl			

Pesticides like organochlorines, organophosphates, carbamates, pyrethroids and anilines can cause acute and chronic health effects, including stinging eyes, rashes, and death, while chronic effects include cancers [100], [164], birth defects, reproductive harm [44], neurological effects [2] and endocrine system disruption [45]. Since chronic effects of pesticide exposure may not appear for weeks, months, or even years after exposure, it can be difficult to link exposure to harmful health effects. Studies on leukaemia, lymphoma, and brain tumours in humans have been connected to Nicotine, DDT, 2,4- dichlorophenoxyacetic acid [14], [130], [164]. Hormone-like substances called endocrine disruptors can seriously impair both human and animal health by interfering with physiological processes. Exposure of humans to these chemicals has been linked to comparable effects [158].

With the abundance of research on the health effects of pesticide poisoning, a thorough analysis that concentrates on the unique health consequences linked to different chemical groups of pesticides has been organised as follows (Table 5):

### 5.1 Silent hazards of organochlorines

OC are toxic and persistent in the environment; they are categorised as persistent organic pollutants [124]. Major organo-chlorines like DDT, aldrin, endosulfan, endrin, chlordane, mirex, and dieldrin are used for their effectiveness [140].

Organochlorine pesticides (OCPs) like DDT have toxicological properties that are influenced by their physicochemical features and impact on the central nervous system (CNS) and Peripheral nervous system (PNS) [144]. Cyclodienes, hexachlorocyclohexane, endosulfan and lindane can easily pass through the skin, so they are easily found in human tissues like blood, breast milk, and fatty tissues and have been linked to chronic illnesses like Parkinson's disease [32]. Symptoms including headaches, vomiting, nausea, headache, nausea, dizziness, vomiting, tremors, lack of coordination, mental confusion and mental disorientation can be brought on by exposure to OCPs [144]. They can interfere with the endocrine system, increase the risk of hormone-related cancers, and be neurotoxic and carcinogenic [110]. Generally, they induce either central nervous system depression or stimulation, depending on the specific pharmaceutical substance and dosage. Acute organochlorine poisoning causes rapid onset of central nervous system excitement and depression, resulting in symptoms like euphoria, hallucinations, agitation, unconsciousness, seizures, and additional respiratory, gastrointestinal, and nervous system symptoms [93].

OCPs, DDT and DDE can interfere with lactation during pregnancy due to their estrogenic properties. The weak estrogens function in conjunction with the endogenous estrogens, affecting the endocrine system and causing damage to female reproductive physiology, particularly lactation. Estrogen stimulates the pituitary gland, leading to increased Prolactin (PRL) levels [3]. Additionally, OCPs can compete with prolactin receptors to block PRL's lactation-inducing effect. Research demonstrates that OCPs can cause Ca_2_^+^ influx, which in turn causes PRL secretion and compromises endocrine function [170]. Additionally, OCPs have an impact on lactation by activating aryl hydrocarbon receptor, which impairs the differentiation of the mammary glands during pregnancy.

Because of their higher body surface area and immature metabolic pathways, foetuses and infants may be more sensitive to oral contraceptives (OCPs) than adults. When OCPs with a molecular weight less than 800 D passively enter breast tissue, the physical burden of oral contraceptives in children is increased. Breastfeeding exposes children before the age of seven years to early life exposure, which greatly increases the physical burden of contraceptives and is a major factor in determining blood levels [11]. OCP exposure after delivery has an impact on the growth and development of the infant and may raise the risk of childhood obesity. Children under six years have an increased risk of obesity due to prenatal exposure to HCB [150]. Endocrine disruptors in breast milk can affect the infant's gut microbiome, affecting its composition and function. At this point, exposure to OCP may raise the risk of breast cancer, and long-term OCP exposure in a mother's breast milk may have a deleterious effect on a boy's testicular descent [26]. Postpartum breastfeeding and exposure to endocrine disruptors (OCPs) can also impact infant neurodevelopment. cis-heptachloroepoxide disrupts the dopamine system and negatively impacts the Mental Development Index at 18 months[175].

Exposure to organic compounds (OCPs) in breast milk can negatively impact infants' neurological function. The exact mechanism behind these effects is unclear, but several possible explanations include inhibiting GABA receptors, disrupting thyroid hormones, disrupting calcium signalling, activating PPARs, and lipid metabolism. DDT and its metabolites can contaminate breast milk, causing neurodevelopmental toxicity [167].

OCPs pose a significant threat to aquatic life, disrupting reproductive systems, immune functions, and metabolic functions. Research shows they affect early fish development, disrupt neuroendocrine pathways, and activate estrogen, androgen, and retinoic acid receptors with high affinity. Despite being banned, it's crucial to assess risks for OCP exposure, as these long-lasting, persistent chemicals can bioaccumulate to toxic levels [91].

In order to reduce your exposure to OCPs, you can:

• Substitute less hazardous options for OCPs
• Re-examine human activity, goods, and production methods.
• Educate and inform the general public

### 5.2 Silent hazards of organophosphates

There are several ways that people can come into contact with organophosphates, such as working on farms, handling pesticides, and consuming certain foods and beverages. Organophosphate pesticides cause SLUDGE, characterized by symptoms like salivation, lacrimation, urination, defecation, gastric cramps, and emesis, due to cholinergic overstimulation. Extreme cases may cause muscle fasciculations, diaphoresis, and unresponsiveness [110].

As previously noted, DUMBELS is a commonly used mnemonic that incorporates the muscarinic consequences of organophosphate poisoning, Diaphoresis/defecation, urination, miosis, bronchospasm/bronchorrhea, emesis, lacrimation, and salivation. OPs cause the body to produce an excess of ACh by inhibiting AChE. Cholinergic toxidrome, respiratory failure, and neurological problems are the outcomes of this. The AChE enzyme is cleaved by organophosphate, which makes it inactive. Though it happens more slowly than inhibition, regeneration is possible. The connection becomes irreversible as the enzyme ages [127].

OP exposure can cause acute toxicity, leading to high morbidity and even death. The toxicity depends on environmental exposure level, dose absorbed, and individual ChE depression. Public health is at risk from long-term exposure to low to moderate levels of organophosphates, particularly agricultural and pesticide manufacturing workers. Long-term effects are of interest to researchers, as chronic neurological sequelae have been reported [66].

OPs, with their large distribution and lipophilic characteristics, quickly spread into the liver, kidneys, and adipose tissue, providing defence against metabolism. Post-poisoning, lipophilicity and adipose tissue can impact outcomes. A 2014 Korean study found that higher BMI (body mass index) was associated with longer mechanical breathing, ICU stays, and overall hospital stays in obese patients (D. H. [79]).

A 22-year-old woman in her 29th week of pregnancy presented with tonic-clonic seizures, indicating OP poisoning. Initially diagnosed as eclampsia with intravenous magnesium sulphate treatment, she developed symptoms of OP toxicity, leading to intravenous atropine treatment. The baby was born prematurely and died two days later, highlighting the potential of OP poisoning in pregnancy complications. This instance underlines the possibility of OP poisoning in a study [147]

Studies conducted on male agricultural workers reveal reduced levels of luteinizing hormone and serum testosterone as a result of pesticide (OP) exposure. OPs have the potential to disrupt the hypothalamic-pituitary endocrine system, which could impact male hormones such as FSH and LH. Negative impacts on the female reproductive system caused by exposure to OPs include irregular menstrual cycles, lower fertility, prolonged pregnancy durations, spontaneous abortions, stillbirths, and anomalies in development [114].

A 51-year-old farmer was admitted to the hospital after consuming an unknown quantity of parathion, despite having been treated for six years with oral hypoglycemic medicine. The toxin was detected after gastric lavage, leading to a myocardial infarction and death [72].

Worldwide, organophosphate insecticide-related death rates vary from 2 % to 25 %. The pesticides dichlorvos, trichlorfon, malathion, and fenitrothion are the most commonly linked to mortality. The primary cause of death is respiratory failure [77].

### 5.3 Silent hazards of carbamates

Carbamate insecticides are made from carbamic acid, same like OPs are from phosphoric acid. Carbamate poisoning cases are primarily caused by intentional ingestion/dermal exposure, with large outbreaks in developing countries. Rapid symptom onset can occur from combined dermal and inhalational exposures. Carbamates can be chronic or acutely absorbed from various body parts, with low dermal absorption and increased absorption in skin disruption and highly toxic exposure.

Carbamate pesticides target pests' nervous system, inhibiting cholinesterases enzyme activity, causing symptoms like breathing difficulties, muscle weakness, twitching, and severe cases, respiratory paralysis, seizures, and coma. Similar to organophosphates, carbamates are pesticides that cause (AChE) at synapses and junctions to be carbamylated. Their reversible binding to (AChE) results in toxicological manifestations akin to those of organophosphate poisonings, usually subsiding in less than a day. Because of their reversible AChE inhibition, CMs are thought to be significantly less hazardous than OPs, which are extremely harmful irreversible AChE inhibitors [142].

Kitamura et al., [74] detailed the endocrine disruption caused by pesticides like OP and CM, which interferes with male and female reproductive processes. Adult hormones control physiological processes, but pesticides causing endocrine disruption have more detrimental impacts on foetuses and developing creatures. These pesticides affect gene expression [88], organ development [52], and long-term hormonal "set points," such as receptor counts and hormone synthesis [99].

A study in France between 2012 and 2021 found that most attempted suicides linked to Aldicarb had pathognomonic symptoms, with 58.3 % having muscarinic syndrome, 23.3 % nicotinic syndrome, and 61.7 %) central nervous system damage. Two people died and 73.3 % required hospitalization. Blood cholinesterase activity was used for diagnosis, with 87.5 % of cases showing a significant drop in butyrylcholinesterase activity. Quantification of aldicarb and its metabolites is unlikely to change emergency medical care [81].

Chemical toxicants, such as OPs/CMs, can disrupt/damage the endocrine system by affecting the transport, synthesis, binding, secretion, and elimination of natural hormones, which are crucial for homeostasis, reproduction, development, and behavior. Ops/CMs pesticides can also disrupt the pituitary-testicular axis or pituitary-adrenal, leading to persistent endocrine dysfunction [52].

### 5.4 Silent hazards of pyrethroids

About 1800, pyrethrum—a naturally occurring mixture of chemicals found in chrysanthemum flowers—was discovered to have insecticidal effects in Asia. It contains six active pyrethrins, which kill ticks and insects. Pyrethroids are chemical compounds similar to pyrethrins but are more toxic/harmful to insects and mammals, and persist longer in the environment. Pyrethrins and pyrethroids are frequently combined with synergists, which enhance their insecticidal activity by preventing enzymes from breaking down them, increasing their toxicity [13]. Headache, vomiting, facial paraesthesia, dizziness, nausea, burning sensation, muscle fasciculations, skin itchiness, loss of consciousness, low energy, altered awareness, and convulsions are some of the symptoms of pyrethroid pesticide poisoning [132]. Pyrethroids can disrupt the immune system and cause neuro-behavioural effects with chronic exposure. Pyrethroids disrupt electrical signalling in the nervous system by modifying sodium channels in neuronal membranes, leading to symptoms like excitation and convulsions when ingested orally [124].

Research from China and Bolivia indicates a strong correlation between aberrant glucose regulation and cumulative pesticide exposure. Children who have elevated metabolites are twice as likely to develop acute lymphocytic leukaemia [123].

A study of 2116 US adults found that an increased risk of mortality from cardiovascular and all-cause diseases was linked to environmental exposure to pyrethroid insecticides. The study included participants of various ancestry and found that higher urinary 3-phenoxybenzoic acid levels were associated with higher mortality rates. The findings suggest that further research is needed to replicate these findings and understand the underlying mechanisms behind these health risks. Because they are effective against insects and have minimal acute toxic effects on mammals, pyrethroid insecticides are widely used. However, chronic exposure to pyrethroids remains unknown. According to epidemiological research, it may disrupt neurodevelopment, impede fertility, and raise the risk of long-term conditions like diabetes and heart disease [10].

A 12-year-old Afghan child was exposed to pyrethroid chemical permethrin poisoning. She had no history of illness, however she was in a comatose state due to central diabetes insipidus and bilateral mydriasis. Although treated with 10 % permethrin without organophosphates, according to chemical analysis, she passed away after seven days from severe brain injury and resistive hypotension [9].

### 5.5 Silent hazards of anilides/anilines

According to [6] anilines are organic compounds that are utilised in the manufacturing of agricultural chemicals, insecticides, and herbicides. They may irritate the respiratory system, skin, and eyes, which may result in methemoglobinemia, a condition that reduces the amount of oxygen that reaches tissues. Haemolytic anaemia can occur suddenly or gradually as a result of the breakdown of red blood cells. Haemolysis may have subsequent consequences on the kidneys, liver, and heart. Anaemia, tremor, cardiac arrhythmia, pain, narcosis or coma, headaches, and parathesis can all result with prolonged exposure to aniline. Damage to the liver, kidneys, and heart might result from haemolysis as a side effect. Given their prolonged latency period, children might be more serious. Aniline may also have an impact on the nervous system, leading to agitation, exhaustion, somnolence, convulsions, sore muscles, and even unconsciousness [93].

A 25-year-old male patient was referred to the hospital after consuming 200 mL of pretilachlor 50 % EC three hours prior. Upon arrival, he experienced nausea, vomiting, and abdominal pain. Despite his incontinence, he had regular bowel movements and a normal radial pulse. An another case of a 21-year-old female with a history of ingestion of pretilachlor 50 % EC herbicide reported abdominal pain, lacrimation, vomiting, and salivation. Despite her symptoms, she had systemic examinations and no peculiar garlicky smell in her breath. These studies concludes that patients with pretilachlor poisoning frequently have symptoms similar to cholinergic toxicity, such as nausea, vomiting, diarrhoea, sleepiness, stupor, seizures, and fever [29].

### 5.6 Silent hazaRds Of Triazines

Herbicides with triazine bases, glyphosate, dicamba, sodium chlorate, and sodium arsenite are the most widely used ones. Triazines are a class of pesticides that can be toxic to humans, and can cause a variety of health issues. Chronic exposure to atrazine can increase the danger of thyroid, breast, and prostate cancer due to its hormonal effects [148].

The Environmental Protection Agency has issued a lifetime health advisory limit of 3 microgrammes per litre for atrazine, which is regarded as mildly to moderately detrimental to humans. It is acceptable to drink water that has atrazine levels below this [146]. Excessive atrazine consumption in animals can cause tremors, organ weight changes, and liver and heart damage. Farmers exposed to atrazine have a marginally increased risk of non-Hodgkin's lymphoma [23]. Triazine/atrazine exposure weakly correlates with ovarian, breast, and prostate cancers [111].

Given that atrazine alters hormones, epidemiological studies that link the herbicide's exposure to reproductive effects— a higher chances of miscarriage, decreased male fertility, an increased risk of any birth defect, low birth weight, and a higher incidence of abdominal defects—are not surprising [111].

## 6 Pesticide exposure and diseases

Exposure to pesticides has emerged as a major public health concern due to mounting data that associates it with various harmful health outcomes and illnesses. The use of chemical pesticides and more intensive agricultural practices necessitates an awareness of the possible dangers connected with these compounds. Pesticides are not only associated with life-threatening diseases like cancer, but also with various other disorders that can be fatal if untreated, compromising an individual's quality of life. Studies have indicated links between contact to pesticides and a range of health concerns, i.e, neurological illnesses, respiratory ailments, difficulties with reproductive health, and even specific forms of cancer[77]. In order to protect human health and wellbeing, this overview tries to highlight the many health effects of pesticide exposure. It also emphasises the significance of taking precautions and using pesticides responsibly. Here are some of the key conditions linked to pesticide exposure:

### 6.1 Neurological disorders

**Fig. 5:**
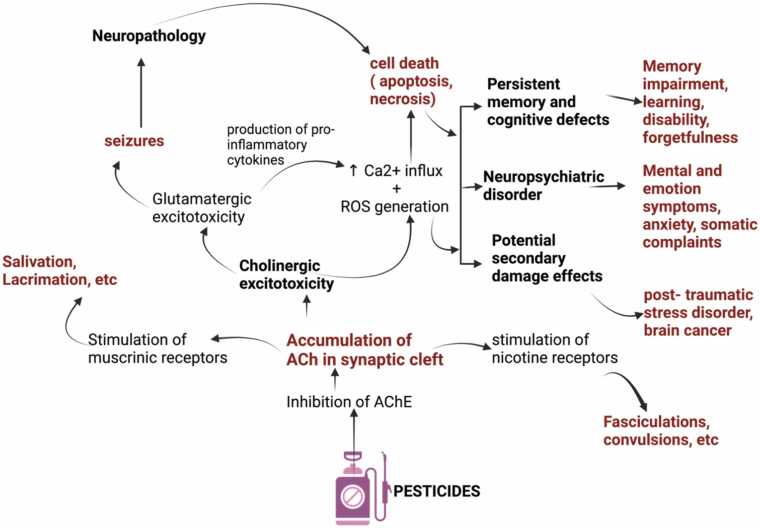
Mechanism of pesticide toxicity on the nervous system [28], [98], [130].

Pesticides can affect the neurological system in a number of ways, which can result in a variety of neurological conditions. Numerous insecticides, particularly carbamates and organophosphates, have neurotoxic properties. They prevent the breakdown of the neurotransmitter ACh in the synaptic cleft by inhibiting the enzyme AChE. ACh builds up when AChE is blocked, which causes cholinergic receptors to become overstimulated. The overstimulation can cause symptoms like paralysis, jerking of the muscles, and respiratory failure [28]. ROS, which can harm lipids, proteins, and DNA in cells, is one way that pesticides can cause oxidative stress. Neurodegenerative illnesses like Parkinson's and Alzheimer's have been linked to this oxidative damage, which can result in the death of neural cells (Fig. 5).

**Parkinson's disease**: Parkinson's disease (PD) is a neurological condition causing stiffness, tremors, and balance issues, making daily tasks more challenging for individuals. The cause of PD is unknown, but environmental factors like pesticides, such as rotenone, have been linked to its initiation. Certain pesticides, especially organophosphates and carbamates, have been linked to an increased risk of developing Parkinson's disease. Chronic inhibition of mitochondrial complex I by rotenone causes selective nigrostriatal dopaminergic degeneration, leading to partial or complete muscle movement due to disruption in the basal ganglia [130].

A meta-analysis of 104 studies found that PD was linked to farming and pesticides, particularly in self-reported cases. Heterogeneity in risk estimates for specific exposures was discovered to be caused by the quality of the studies, though. Good case-control studies demonstrated that solvent, herbicide, and pesticide exposure raised the risk of PD. Heterogeneity remained high for farming, pesticides, organochlorines, and organophosphates in high-quality case-control studies, with a notable risk associated with living in rural areas [116]. Paraquat and rotenone cause oxidative stress and mitochondrial dysfunction, contributing to nerve cell death in Parkinson's disease, highlighting the common themes in understanding cell death [47].

**Cognitive impairment:** Long-term exposure may lead to memory and cognitive function issues. Pesticide-induced brain injury affects neurotransmitters, including choline, free amino acids, and monoamines. Choline substances, such as ACh and AChE, form the central acetyl cholinergic neuron system. ACh is synthesized in the presynaptic membrane and binds to the cholinergic receptor of the postsynaptic membrane to form acetylase (HAT). However, pesticides like monocrotophos (MCP) and chlorpyrifos (CPF) can inhibit AChE's activity, leading to CNS toxicity, neural signalling disturbances, and cognitive impairment [98].

In the cerebral cortex and hippocampus, free amino acids like glutamic acid (Glu) and N-methyl-D-aspartic acid (NMDA) are essential for learning and memory formation. They participate in the transmission of synaptic excitation. They are classified as inhibitory amino acids (IAAs) like γ-aminobutyric acid (GABA) and Gly, and excitatory amino acids (EAAs) like glutamic acid (Glu). Pesticides such as atrazine and chlorpyrifos cause abnormal fluctuations in their levels within the organism, which interfere with nerve signal transduction and impact the synthesis and regulation of related proteins involved in memory and learning [19].

Dopamine (DA), norepinephrine (NE), and 5-hydroxytryptamine (5-HT) are examples of monoamine neurotransmitters that control movement, emotion, sleep, thought, and memory. Pesticides such as Amitraz, Profenofos, and Paclobutrazol have the ability to decrease the amount of monoamine neurotransmitters expressed in brain tissue [172]

Despite growing evidence that links pesticide exposure to neurological diseases, epidemiological data regarding the neurobehavioral effects of chronic pesticide exposure are scarce. As part of the Swedish study Prospective Investigation of the Vasculature in Uppsala Seniors (PIVUS), the plasma concentrations of three organic compounds (p,p′-DDE, trans-nonachlor, and hexachlorobenzene) were measured in 989 men and women aged 70 years. The results showed that those with high OC levels were roughly three times more likely than people with low levels of OC to experience cognitive impairment in the future [80].

**Alzheimer’s disease:** Alzheimer's disease (AD) is a progressive neurological disorder characterized by irreversible neuronal loss. The main aggregated proteins involved in AD are amyloid plaques and neurofibrillary tangles, which lead to synapses loss, neuronal cell death, and cognitive dysfunction. These neuropathological changes are thought to be the main cause of AD. Pesticide exposure has been linked to Alzheimer's disease (AD), a neurodegenerative disorder strongly linked to the disease. As neurotoxins, pesticides are linked to a number of neurodegenerative conditions, such as dementia and mild cognitive impairment. Research has indicated a favourable correlation between pesticide exposure and mild cognitive impairment, indicating that regular exposure to pesticides may increase the risk of developing AD. Chronic or occupational pesticide exposure has also been recognized as a risk factor for dementia. According to research conducted in vitro, pesticides have the ability to elevate levels of BACE1 and amyloid-β precursor protein, which can impede the removal and extracellular breakdown of amyloid-β peptides. According to in vivo research, pesticides may interfere with metabolic pathways that maintain amyloid-β homeostasis, which would raise amyloid-β levels, memory loss, and reduced motor activity (G. Salazar et al., 2011). The study found a positive association between pesticide exposure and Alzheimer's disease (AD), with a 3.8-fold increase in organochlorine metabolites in AD patients compared to control participants [145]

### 6.2 Respiratory problems

According to a study, lung function declines and a higher severity of COPD is diagnosed in 10.9 % of agricultural workers. Agricultural workers have lower red blood cell AChE levels, which are positively correlated with respiratory symptoms, a decline in lung function, and COPD. These health problems are associated with prolonged exposure to cholinesterase-inhibiting agricultural pesticides in India [18].

**Asthma:** A Spanish study found that exposure to pesticides, particularly DDE and polychlorobiphenyls, can worsen asthma symptoms and increase the risk of developing asthma. The study involved 405 children and 482 mothers and found a strong correlation between these chemicals and asthma [86].

Pesticide aerosols can damage bronchial mucosa cells or interact with airway receptors to cause neurogenic inflammation. Inflammatory cytokines and neuropeptides from sensory neurons release inflammatory mediators, causing tissue damage and airway inflammation [56]. High pesticide exposure events were linked to a doubling of both allergic and nonallergic asthma, according to a study involving 19,704 male farmers. Four pesticides were linked to nonallergic asthma and twelve pesticides were linked to allergic asthma. DDT had the strongest correlation with nonallergic asthma, but there was no indication that usage would cause asthma to worsen [59]

**Chronic obstructive pulmonary disease (COPD**): Some studies suggest a link between pesticide exposure and COPD. A large study of nearly 100,000 people in the UK found that workers exposed to pesticides had a higher risk of developing COPD. The study found that workers with sustained, high-intensity exposure had a 32 % higher risk, while those exposed at any point had a 13 % higher risk. The study also identified agriculture, fishing, and grounds keeping as occupations with a higher risk of COPD [68]. While there is less evidence for COPD than there is for asthma, exposure to pesticides is more strongly linked to asthma in children [37].

### 6.3 Reproductive health issues

**Fig. 6:**
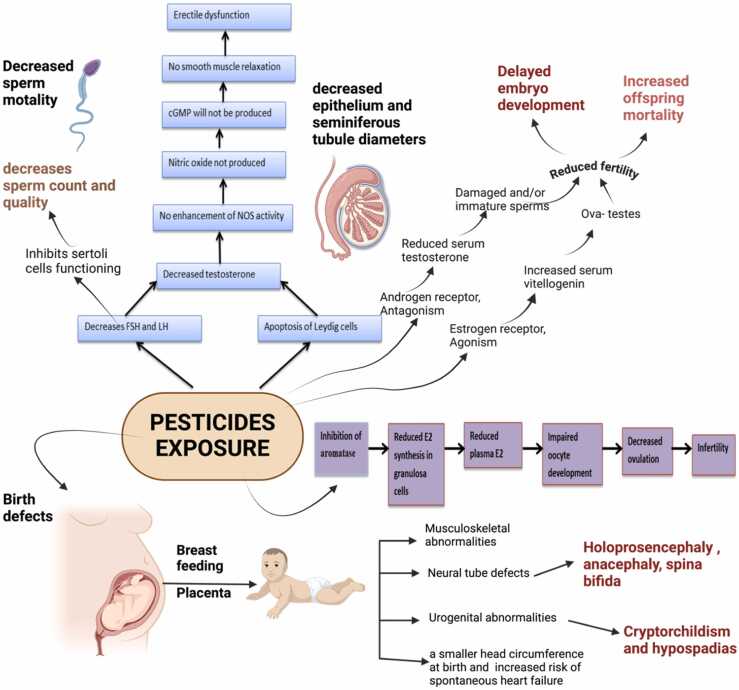
Mechanism of pesticide’s toxicity on reproductive health [115] (Sampaio et al., 2020) (Wu et al., 2023) [155].

Scientists now know that pesticide exposure can cause a variety of reproductive problems that can affect children, adults, and men equally (Fig. 6). The risk of reproductive and developmental problems, such as aberrant sperm, reduced fertility, and birth malformations, can rise with pesticide exposure. Breast milk may potentially be contaminated by pesticide exposure in the workplace or other environmental conditions. Exposure to mixed pesticides can have negative consequences, particularly if personal protection equipment is not worn. In poor nations, toxic pesticides are still widely used. Assessing exposure levels, balancing risks and benefits, and implementing strategies to lower absorbed amounts are all part of providing patients with pesticide impacts counselling [44].

**Birth defects:** Pregnant women exposed to certain pesticides (e.g., glyphosates, paraquat, imidacloprid etc) may have a higher risk of giving birth to children with congenital anomalies. Exposure to pesticides during pregnancy can cause problems like miscarriage, birth defects, and fetal growth retardation. From 2002 to 2006, researchers looked into how exposure to agricultural pesticides in the environment during pregnancy affected the growth of the foetus in Brittany. The findings indicated that newborns born to mothers who lived in rural municipalities had a smaller head circumference and were more likely to suffer from spontaneous heart failure (SHC) [115]. The reproductive system of male progeny is impacted by exposure to OPP during gestation and lactation, leading to abnormalities in testicular development and morphology. Male offspring exhibit increased tubule numbers and decreased epithelium and seminiferous tubule diameters, despite the fact that maternal exposure has no effect on testicular function [133].

**Fertility problems:** Infertility is the failure to conceive. Some pesticides have been associated with reduced fertility in both men and women. The study examined the association between self-reported infertility among American women aged 20–50 and four distinct metabolites of single organophosphate pesticides (OPPs) and their mixed exposure. The findings demonstrated a strong positive correlation between the mixed OPP metabolites and infertility, with dimethylphosphate (DMP) being the OPP metabolite that most significantly increases the risk of infertility in females [171].

**Sperm:** Pesticides can cause sperm abnormalities, including low concentration, reduced motility, and abnormal morphology. According to recent research, working with pesticides increases the likelihood that sperm will exhibit morphological abnormalities, such as a decrease in the quantity and percentage of viable sperm. Occupational pesticides like parathion and methyl parathion can decrease sperm concentration and affect sex accessory glands, reducing seminal volume and increasing abnormal sperm head morphology. Because organochlorine OCPs, like endosulfan and DDT, directly interact with sex steroid receptors and disrupt the hypothalamic-pituitary-testes axis, they specifically negatively impact the male reproductive system. Phthalates have also been found to have adverse effects on sperm quality, with sperm motility decreased and cytotoxicity caused [141]. Carbosulfan, a potent genotoxic pesticide, increases bone marrow micronucleus formation, sperm abnormality in mice, and chromosomal aberrations, indicating potential germ cell mutagen effects [48]. Studies show a link between 3-PBA metabolites in human residues and reduced sperm concentration, increased sperm DNA fragmentation, and decreased sperm motility [155].

A study presents epidemiological research that show a correlation between parents' occupation in agriculture and the following outcomes: low birthweight, small-for-gestational-age (SGA) births, preterm delivery, stillbirth, congenital abnormalities, and miscarriages. The analysis' findings demonstrated that working in agriculture raises the possibility of certain sperm morphological anomalies, such as a drop in the number of viable sperm and sperm count per ejaculate. Pesticide exposure generally did not appear to have any influence on sexual hormones. Unambiguous statistics exist on the impact of working in agriculture on the time it takes to become pregnant, but the majority of them imply a connection between pesticide exposure and a lower fecundability ratio. Furthermore, there are no clear findings from studies on the sex ratio of kids [54].

### 6.4 Cancer

Exposure of various persticides like DDT, Dichlorimethane, Diazinon, Non-arsenical insecticides, Polychlorinated biphyenyls, 1,3-Butadiene Pentachlorophenol, Chlorophenoxy herbicides, Malathion are linked to increased risk of various cancers in both children and adults, including leukemia, Burkitt lymphoma, neutrophiloma, Wilm's tumor, non-Hodgkin lymphoma, soft tissue sarcoma, Digestive organ cancer, Respiratory cancer, Breast, Male and Female genital organ cancer [63].

**Non-hodgkin lymphoma:** Non-Hodgkin lymphoma (NHL) is the fifth most common cancer globally, primarily caused by viral and bacterial infections. Environmental factors, such as farming, agricultural chemicals, and pesticides, have been linked to increased NHL risk, with organophosphate pesticides being a recent substitute [60]. Several studies have found a connection between pesticide exposure and an increased risk of non-Hodgkin lymphoma. Exposure to Chlorophenoxy herbicides, DDT, Diazinon, Phenolic acid herbicides, specifically 2,4-dichlorophenoxyacetic acid, in agricultural settings has been associated with non-Hodgkin lymphoma, a worldwide cancer [63]. Research indicates a 2–8-fold heightened risk of NHL, with immunological dysfunction being a direct cause of NHL [130].

**Leukemia:** Leukemia, a blood cancer originating from bone marrow, produces abnormal blood cells, affecting normal blood cell production, causing bleeding, bruising, bone pain, fatigue, fever, increased infection risk, weight loss, and abdominal pain. Pesticide exposure is a significant cause of acute leukemia [63]. Certain pesticides, particularly organophosphates, have been linked to an elevated risk of childhood leukemia and studies have shown that exposure during pregnancy increases the risk of lymphoma and leukemia in children. Nicotine, DDT, and certain animal insecticides raised the risk of leukaemia by ≥ 2.0 [14]. The meta-rate ratio estimate for cohort studies showed significant heterogeneity, with an increased risk of acute myeloid leukemia in manufacturing workers and pesticide applicators, with no significant heterogeneity found among case-control studies [163].

### 6.5 Endocrine disruption

For their potential to affect human populations' health, especially the reproductive system, endocrine-disrupting chemicals, or EDCs, have been the subject of extensive research. Given their structural diversity and low detection limits, EDCs are difficult to estimate human exposure to. Enzyme and receptor-mediated chemicals, or EDCs, are harmful because they obstruct the regular hormonal homeostatic processes that support tissue growth and development. They can trigger an agonistic effect or an antagonistic action, depending on the receptor they target [102]. Moreover, EDCs affect the enzymes responsible for hormone metabolism and steroidogenesis. One class of pesticides that has anti-androgenic properties is phthalates; they work by preventing Leydig cells from synthesising testosterone [43]. EDCs, found in both in vitro and animal research, affect hormone-dependent pathways, leading to reproductive system disorders like infertility, endometriosis, breast cancer, and low sperm quality, causing oxidative stress and cell death.

**Diabetes:** According to newly available scientific data, diabetes is a side effect of being around pollution. Pesticide exposure, especially that of organochlorines and their metabolites, may increase the risk of type 2 diabetes and its complications. Other studies have also suggested biological mechanisms for the link between OPs and DM, such as chronic exposure to OPs causing increased body weight, disrupting fat and glucose homeostasis, and mediating damage to pancreatic β cells, insulin resistance, and excessive hepatic gluconeogenesis [177].

In order to better understand the relationship between OCPs and the risk factors for type 2 diabetes mellitus (T2DM), such as insulin resistance, lipid metabolism, and glucose intolerance, a study was done on the trend of OCP levels in drinking water and blood samples from the North Indian population. The findings implied that after exposure, high OCP levels may raise the risk of T2DM [161].

**Fig. 7:**
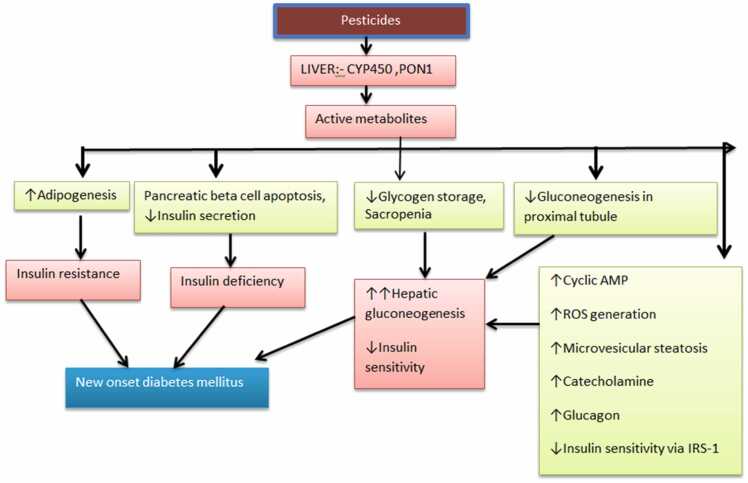
Mechanism of pesticides toxicity causing Diabetes [161], [177].

Other mild disorders have also been observed i.e, Direct contact with pesticides can cause skin rashes, irritation, and allergic reactions. Acute exposure can lead to gastrointestinal symptoms such as nausea, vomiting, and abdominal pain. Some studies suggest a potential link between pesticide exposure and chronic fatigue syndrome. It's important to note that the degree of risk is dependent on various factors, including the type of pesticide, level of exposure, duration of exposure, and individual susceptibility. Protective measures, such as using personal protective equipment (PPE) and following safety guidelines, are crucial for minimizing risks associated with pesticide exposure (Fig. 7).

## 7 Conclusion

Pesticides can be classified based on their nature, chemical composition, mode of entry, and mode of action. The food chain, the ecosystem, and agriculture are all significantly impacted by pesticides, causing high morbidity and mortality rates. They can cause oxidative stress, disrupt endocrine systems, and modulate gene expression, leading to various health issues, including cancer. Poisoning with pesticides is common in India, and aggressive resuscitation and antibiotic use are crucial to reduce mortality. Sensitizing physicians to advances in diagnosis and management is essential. Stricter regulations and sustainable agricultural practices are needed to mitigate their adverse effects. Bioremediation methods like phytoremediation, myco-remediation, bacterial pesticide degradation and microalgae bioremediation can help minimize contaminant hazards on the ecosystem and human. However, there is a lack of information on implementing preventive measures in occupational exposures and evidence-based guidelines for therapeutic interventions following poisoning. Further research is needed to build less harmful, and efficient pesticides for mindful utilization.

## Funding

Not applicable

## CRediT authorship contribution statement

**Chander Shekhar:** Writing – original draft, Data curation, Conceptualization. **Reetu Khosya:** Writing – original draft, Conceptualization. **Amit Kumar Sharma:** Writing – review & editing, Validation, Supervision, Conceptualization. **Kushal Thakur:** Methodology. **Danish Mahajan:** Validation, Software. **Rakesh Kumar:** Writing – review & editing. **Sunil Kumar:** Supervision.

## Declaration of Competing Interest

The authors declare that they have no known competing financial interests or personal relationships that could have appeared to influence the work reported in this paper.

## Data Availability

No data was used for the research described in the article.
